# NLRP3 promotes allergic responses to birch pollen extract in a model of intranasal sensitization

**DOI:** 10.3389/fimmu.2024.1393819

**Published:** 2024-06-12

**Authors:** Renate Bauer, Hieu-Hoa Dang, Daniel Neureiter, Michael Stefan Unger, Theresa Neuper, Melanie Jensen, Alice Emma Taliento, Helen Strandt, Iris Gratz, Richard Weiss, Angelika Sales, Jutta Horejs-Hoeck

**Affiliations:** ^1^ Department of Biosciences and Medical Biology, Paris Lodron University of Salzburg, Salzburg, Austria; ^2^ Center for Tumor Biology and Immunology, Paris Lodron University of Salzburg, Salzburg, Austria; ^3^ Institute of Pathology, Paracelsus Medical University/University Hospital Salzburg (SALK), Salzburg, Austria; ^4^ Division of Newborn Medicine, Department of Pediatrics, Massachusetts General Hospital, Boston, MA, United States; ^5^ Department of Dermatology, Venerology and Allergology, Medical University of Innsbruck, Innsbruck, Austria

**Keywords:** NLR, NLRP3, inflammasome, birch pollen allergy, intranasal sensitization

## Abstract

**Introduction & Objective:**

Allergic sensitization is an essential step in the development of allergic airway inflammation to birch pollen (BP); however, this process remains to be fully elucidated. Recent scientific advances have highlighted the importance of the allergen context. In this regard, microbial patterns (PAMPs) present on BP have attracted increasing interest. As these PAMPs are recognized by specialized pattern recognition receptors (PRRs), this study aims at investigating the roles of intracellular PRRs and the inflammasome regulator NLRP3.

**Methods:**

We established a physiologically relevant intranasal and adjuvant-free sensitization procedure to study BP-induced systemic and local lung inflammation.

**Results:**

Strikingly, BP-sensitized *Nlrp3*-deficient mice showed significantly lower IgE levels, Th2-associated cytokines, cell infiltration into the lung, mucin production and epithelial thickening than their wild-type counterparts, which appears to be independent of inflammasome formation. Intriguingly, bone-marrow chimera revealed that expression of NLRP3 in the hematopoietic system is required to trigger an allergic response.

**Conclusion:**

Overall, this study identifies NLRP3 as an important driver of BP-induced allergic immune responses.

## Introduction

Approximately 20–30% of adults and up to 40% of children worldwide suffer from allergic diseases, and allergies to inhalant allergens such as birch pollen (BP) are increasing in the northern hemisphere (1). Allergic immune reactions are characterized by the induction of a type 2 response including Th2 cell differentiation, secretion of the major cytokine IL-4 and release of the antibody subtype IgE. This in turn mediates symptoms such as allergic rhinitis, rhinoconjunctivitis and in severe cases allergic asthma (2, 3). In Germany, 14.1% of children (aged 3–17 years) were found to be sensitized to the major BP allergen Bet v 1 (4), and 16.3% of adolescents and young adults (aged 12–21 years) in an Austrian study showed IgE reactivity to BP (5). Moreover, in the New York area as well as Canada, a high prevalence (26.8–32.9%) of hypersensitivity to birch was found (6, 7).

Recent scientific advances have highlighted the importance of the whole pollen composition rather than just the major allergen itself. BP, for example, is composed not only of pollen-intrinsic factors such as proteins, lipids and sugars, but also has its own microbiome of several bacterial species, most of them Gram-positive (3, 8–10). It is well established that specific pathogen-associated molecular patterns (PAMPs) like peptidoglycan derived from bacteria are recognized by pattern recognition receptors (PRRs) such as the intracellular NACHT-, Leucin-rich repeat- and Pyrin- domains-containing protein 3 (NLRP3), which may be capable of shaping the allergen-dependent immune response (11).

While NLRP3 has several canonical and non-canonical functions, it is best known for its ability to form an inflammasome together with ASC (Apoptosis-associated Speck-like protein containing a Caspase Recruitment Domain) and Caspase 1 in response to various bacterial or viral stimuli (12, 13). Formation of the NLRP3 inflammasome results in proteolytic cleavage of Caspase 1 and subsequent activation and release of IL-1β and other pro-inflammatory mediators via Gasdermin D pores (14). This NLRP3-inflammasome-induced IL-1 signaling was shown to be required for the induction of an immune response in an ovalbumin-induced model of allergic sensitization (15). However, subsequently, NLRP3 was also described to act as a transcription factor required for Th2-cell differentiation, a function which was completely independent of inflammasome formation (16). Additionally, there are a few reports on house dust mite (HDM)- induced allergy models which surprisingly found no role for NLRP3 (17, 18). Thus, there is no consensus view on the specific role of NLRP3 in the induction of different allergies. Moreover, although activation of NLRP3 has already been shown to regulate phagosome function and thereby affecting the processing and allergenicity of antigens such as Bet v 1 (19, 20), involvement of NLRP3 in the sensitization to BP has not yet been studied.

Therefore, this study aimed to investigate the role of NLRP3 during allergic sensitization induced by birch pollen extract (BPE). First, we thoroughly describe a novel and more physiological intranasal sensitization model to BPE which potently induces allergic airway inflammation in an adjuvant-free manner. Applying this model, we demonstrate that NLRP3-deficiency partially protects against BPE-induced allergic responses, evidently independent of inflammasome activation and subsequent Caspase 1 activity. Strikingly, bone marrow chimera studies revealed that allergic sensitization to BPE is reliant on NLRP3 expression in the hematopoietic system.

Taken together, this study describes a highly physiological, intranasal sensitization model to study allergic airway inflammation and demonstrates that knockout of NLRP3 in the hematopoietic system partially protects against increased BPE-induced lung inflammation.

## Materials and methods

### Preparation of aqueous birch pollen extract

Pollen from birch (*Betula pendula*) were obtained from Allergon (batches: 012518101, 0000468269) and extracted in Dulbecco’s Phosphate Buffered Saline (PBS, Sigma Aldrich) via constant agitation at 600 rpm for 1 hour at room temperature. Protein content was determined after sterile filtration through a 0.22 µm filter using a Pierce™ 660nm Protein Assay (Thermo Fisher Scientific) according to manufacturer’s instructions.

### Mouse lines

C57BL/6 wild-type (WT) mice were initially obtained from Janvier (France). *Nlrp3^-/-^* (B6.129S6-*Nlrp3^tm1Bhk^*/J, strain# 021302, RRID: IMSR_JAX:021302), *Casp1^-/-^* (B6.Cg-*Casp1^em1Vnce^*/J, strain# 032662, RRID: IMSR_JAX:032662) and B6 Thy1.1 (WT^+^, B6.PL-*Thy1^a^*/CyJ, strain# 000406, RRID: IMSR_JAX:000406) mice were imported from The Jackson Laboratories (USA) via the Charles River Laboratories in Germany.

All mouse lines were housed and bred under specific pathogen-free conditions in individually ventilated cages at the local animal facility at the Paris Lodron University of Salzburg (PLUS) with unlimited access to water and food and maintained under a 12-hour light-dark cycle, constant temperature and humidity.

### *In vivo* sensitization and generation of bone marrow chimeras

All animal experiments complied with EU guidelines (Directive 2010/63/EU and Regulation 2019/1010) and Austrian Regulations (TVG2012) and were approved by the Austrian Federal Ministry of Education, Science and Research (permit numbers: 66.012/0015-V/3b/2019, 2021-0.588.488). Both female and male littermates were used for the described experiments. Each data set was pooled from 2–3 individual experiments.

**Figure 1:**
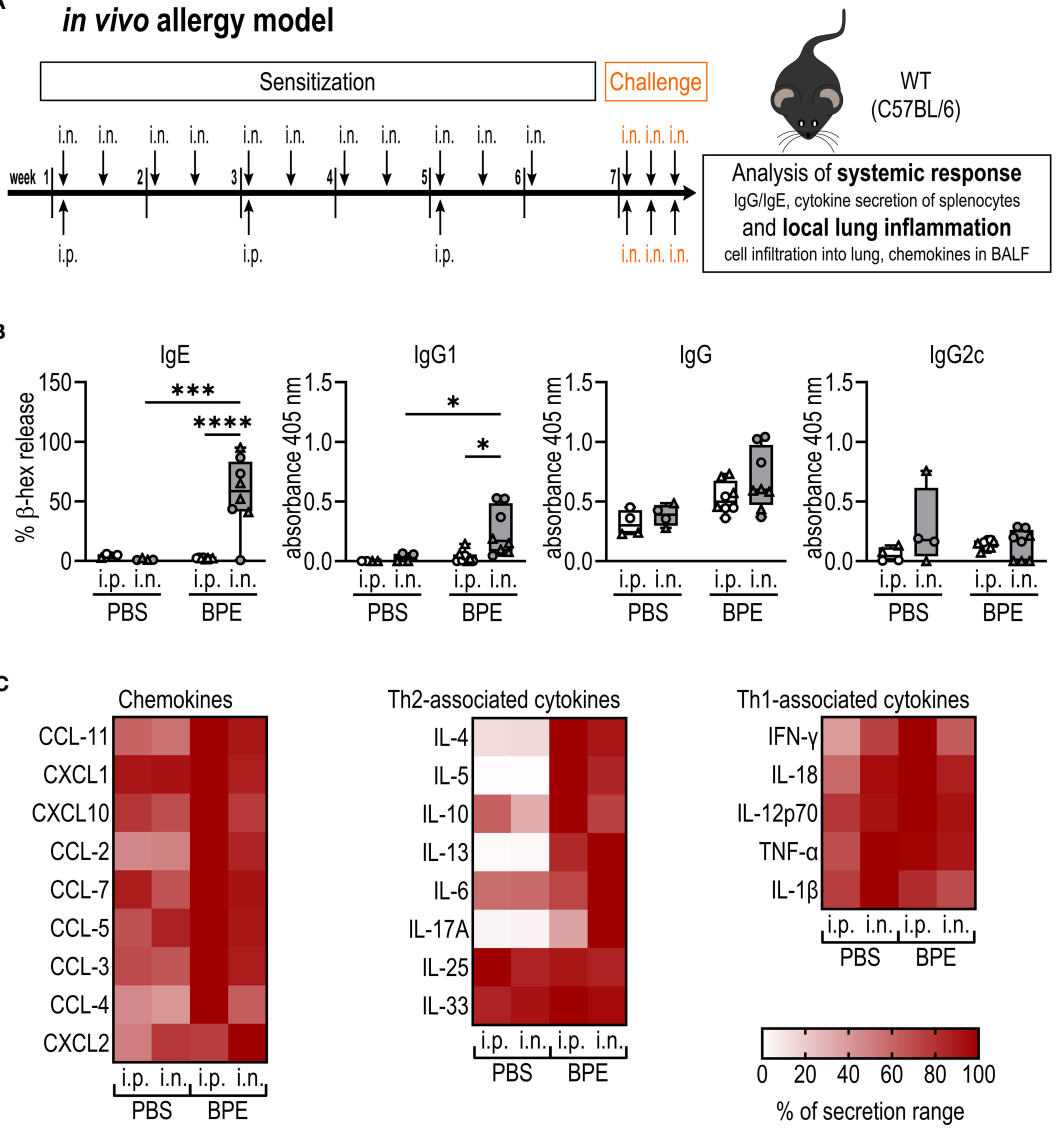
I.n. but not i.p. sensitization induces BPE-specific Th2-associated antibodies. **(A)** Schematic diagram of the allergic sensitization model to BPE. C57BL/6 wild-type (WT) mice were sensitized over a course of 6 weeks either by i.p. injection or i.n. instillation of PBS or 40 µg BPE. In week 7 all mice were challenged i.n. with BPE on 3 consecutive days. One day after the last challenge, mice were euthanized and analyzed for changes in the systemic response **(B, C)** and local lung inflammation (**Figure 2**). **(B)** BPE-specific serum IgE was measured via antigen-induced cross-linking of Fcϵ-receptors on RBL-2H3 cells and subsequent degranulation and release of β-hexosaminidase (β-hex release). Data is displayed as % of maximum release induced by 1% Triton X-100. IgG antibody subtypes specific to BPE were analyzed by ELISA. Box plots show median of 4–8 mice per treatment group of two individual experiments with different batches of pollen extract. Each mouse is depicted as one data point. **(C)** Splenocytes were restimulated with BPE for 3 days and the secreted cytokines analyzed by Luminex multiplex technology. Medians of each group are shown as % of maximum secretion. i.n.=intranasal, i.p.=intraperitoneal, BPE=birch pollen extract. One-way ANOVA with Tukey’s post hoc test was performed to determine statistical significance. *p<0.05, ***p<0.001, ****p<0.0001.

WT, *Nlrp3^-/-^* and *Casp1^-/-^* mice aged 8–14 weeks were sensitized either by i.p. injection of 100 µl PBS or BPE (40 µg protein) for three times every two weeks, or by i.n. instillation of 50 µl PBS or BPE (40 µg protein) under 2.5–3% isoflurane (Iso-Vet) anesthesia for 11 times, twice per week (with intervals of at least 3 days) over a course of 6 weeks. In week 7 all mice were challenged i.n. with 40 µg BPE on three consecutive days. One day after the last challenge, mice were euthanized by cervical dislocation, and the systemic response and local lung inflammation were analyzed (**Figure 1A**).

For bone marrow chimeras, 8- to 12-week-old B6 Thy1.1 (WT^+^, Thy1.1=CD90.1) or *Nlrp3^-/-^* (CD90.2) mice were irradiated twice with 4.75 Gray (9.5 Gray in total) with an interval of 3 hours. Donor bone marrow was isolated from femurs and tibias of WT^+^ or *Nlrp3^-/-^* littermates. After lysis of erythrocytes with Ammonium-Chloride-Potassium (ACK) lysis buffer (150 mM NH_4_Cl, 10mM KHCO_3_, 0.1 mM Na_2_EDTA, pH 7.4) and two washing steps, cells were passed through a 70-µm cell strainer and counted. 1–2 hours after the last irradiation of recipient mice, 5×10^6^ bone marrow cells in PBS were injected intravenously into the tail vein. Mice were provided with a broad-spectrum antibiotic (Baytril, active ingredient: enrofloxacin) in their drinking water and general health status and weight were monitored closely over the first three weeks after irradiation. 10–12 weeks after engraftment, the i.n. sensitization protocol was performed as described above.

### Broncho-alveolar lavage (BAL) and flow cytometry of lung-infiltrating cells and blood

After euthanasia, lungs of mice were lavaged by introducing 2× 1 ml cold PBS into the trachea using a Venflon™ Pro Safety venous catheter (BD) and a syringe. Lavage fluid was screened for secreted cyto- and chemokines via Luminex Multiplex Technology as described below. Cells in broncho-alveolar lavage fluid were blocked with mouse serum and stained with PerCP/Cyanine5.5 anti-mouse CD45 Antibody (clone: 30-F11; Biolegend, RRID: AB_893340), Ly-6G Monoclonal Antibody APC (clone: 1A8-Ly6g, eBioscience, RRID: AB_2573307), CD4 Monoclonal Antibody, eFluor™ 450 (clone: GK1.5, eBioscience, RRID: AB_10718983), CD8a Monoclonal Antibody FITC (clone: 53–6.7; eBioscience, RRID: AB_464916), BD Pharmingen™ PE Rat Anti-Mouse Siglec-F (clone: E50–2440; BD Biosciences, RRID: AB_394341), and PE/Cyanine7 anti-mouse CD19 Antibody (clone: 6D5, RRID: AB_313655, Biolegend), and for bone marrow chimeras additionally with Brilliant Violet 605™ anti-rat CD90/mouse CD90.1 (Thy-1.1) Antibody (clone: OX-7, RRID: AB_2562644, BioLegend) and CD90.2 (Thy-1.2) Monoclonal Antibody APC-eFluor™ 780 (clone: 53–2.1, RRID: AB_1272187, eBioscience). After a washing step with FACS buffer (PBS, 5% (v/v) heat-inactivated FCS (Merck), 3 mM EDTA (Merck)) and ACK lysis, cells were resuspended in FACS buffer. Fluorescence of WT, *Nlrp3^-/-^* and *Casp1^-/-^* cells was measured via BD FACSDiva software and a BD FACSCanto II (BD Biosciences), and infiltration into the lung of WT^+^ or *Nlrp3^-/-^* mice was analyzed using CytExpert software and CytoFLEX S (Beckman Coulter). Gating was performed as described before by Neuper et al (21) except for doublet exclusion. Due to size differences of lymphocytes and granulocytes, single cell gating was performed for each gated population individually. CD90.1 or CD90.2 expression was analyzed within the CD4^+^ T cell group.

Blood was isolated from the heart. 4 µl were added to 20 µl of antibody staining mix diluted in PBS containing 10 mM EDTA. After washing once, lysing erythrocytes with ACK-lysis buffer and two further washing steps, cells were resuspended in PBS/10 mM EDTA and analyzed using CytExpert software and CytoFLEX S.

### Splenocyte restimulation

Spleens were processed and erythrocytes lysed with ACK- lysis buffer. After 2 washing steps with cell culture medium (MEM with Earle’s salts (Sigma Aldrich) supplemented with 1% (v/v) heat-inactivated FCS, 20 mM HEPES (Sigma Aldrich), 4 mM L-Glutamine (Sigma Aldrich), 1 mM Sodium pyruvate (Sigma Aldrich), 100 U/ml Penicillin/100 µg/ml Streptomycin (Sigma Aldrich), 2 µM 2-Mercaptoethanol (Gibco) and 1% (v/v) MEM- non-essential amino acids (Sigma Aldrich)), 1×10^6^ splenocytes were seeded per well to a 48-well plate and stimulated with 10 µg/ml BPE in a total volume of 500 µl for 3 days at 37°C, 5% CO_2_. Supernatants were harvested and secreted cytokines and chemokines were analyzed by Luminex Multiplex Technology.

### Analysis of cytokines/chemokines with luminex multiplex technology

Cytokine and chemokine levels in broncho-alveolar lavage fluid (BALF) and secreted by restimulated splenocytes were measured by a bead-based multiplex technology using ProcartaPlex Mo Cytokine/Chemokine Panel 1A 36plex (Lot #: 304658–002, Thermo Fisher Scientific) and Mouse ProcartaPlex Mix&Match 30-plex (Lot #: 320797–000, Thermo Fisher Scientific). After washing beads with PBS containing 0.05% Tween-20 (Sigma Aldrich), they were resuspended in assay buffer and distributed to a 96-well V-bottom plate. 15 µl/well of standards, BALF or splenocyte supernatant were incubated overnight at 4°C with constant agitation (600 rpm) on an orbital shaker. Subsequently the plate was washed three times with 150 µl/well of wash buffer and incubated with detection antibody solution (15 µl/well) for 30 minutes at room temperature (RT) on an orbital shaker followed by three further washing steps and incubation with 20 µl/well of Streptavidin-PE solution for 30 minutes at RT on an orbital shaker. Afterwards, beads were washed again three times, resuspended in 80 µl reading buffer and measured on a Luminex MAGPIX® instrument (Luminex). Data were evaluated using ProcartaPlex Analyst 1.0 software (Thermo Fisher Scientific).

### Evaluation of serum antibodies

BPE-specific IgG subtypes in serum isolated from heart blood were examined by ELISA. In brief, plates were coated with 4 µg/ml BPE overnight at 4°C. After washing with PBS containing 0.1% (w/v) Tween-20 (PBS/Tween), wells were blocked with 1% BSA (w/v, Sigma Aldrich) in PBS/Tween and subsequently incubated with a serial dilution of sera (1:100 – 1:100,000). After addition of secondary antibodies (HRP Goat anti-mouse IgG Antibody (Biolegend, RRID: AB_315009, 1:2000), Goat anti-mouse IgG1 (γ1) horseradish peroxidase conjugate (Invitrogen, RRID: AB_2534048,1:1000), Goat Anti-Mouse IgG2c heavy chain (HRP) (Abcam, RRID: AB_10680258, 1:5000)), the assay was developed with ABTS substrate (2.2 mg/ml ABTS (Sigma Aldrich) in 0.1 M Citrate buffer (pH 4.25, Bio-Rad), 0.12% (v/v) H_2_O_2_ (Merck)) and absorbance measured at 405 nm using a Tecan infinite® M200 or M200 PRO plate reader.

BPE-specific IgE levels were determined via a cell-based assay using rat basophilic leukemia cells expressing the murine Fcϵ-receptor (RBL-2H3, RRID: CVCL_0591, CRL-2256™, ATCC) as described before (22–25). The adherent cell line was cultured in 70% (v/v) MEM with Earle’s Salts, 20% (v/v) RPMI-1640 (Sigma Aldrich), 10% (v/v) heat-inactivated FCS, 4 mM L-glutamine, 2 mM sodium pyruvate and 100 U/ml penicillin/100 µg/ml streptomycin. No mycoplasma contamination was detected in periodic tests. 8×10^4^ cells were seeded in 100 µl cell culture medium per 96-well. The following day, cells were incubated with mouse sera diluted 1:10 in culture medium for 2 hours at 37°C. After three washing steps with Tyrode’s buffer (Sigma Aldrich) supplemented with 0.1% BSA (w/v), cells were stimulated with 100 µl of Tyrode’s buffer/BSA containing 3 µg/ml BPE for 30 minutes at 37°C. To induce total lysis, 10 µl of 10% Triton X-100 (Sigma Aldrich) was added to control wells. Cross-linking of Fcϵ-receptors upon antigen-binding and subsequent degranulation was determined by means of β-hexosaminidase release. Therefore, 50 µl of cell culture supernatant were transferred to a new plate and incubated with 50 µl of 80 µM 4-Methyl umbelliferyl-N-acetyl-b-D-glucosaminide (Sigma Aldrich) in 0.1 M citrate buffer (pH 4.25) for 1 hour at 37°C. After addition of 100 µl of glycine buffer (0.2 M glycine (Sigma Aldrich), 0.2 M NaCl (Roth), pH 10.7), fluorescence was measured with a Tecan plate reader at an excitation wavelength of 360 nm and an emission wavelength of 465 nm, and β-hexosaminidase release was calculated as percentage of total lysis.

### Histomorphology

Histomorphology of lung specimens was performed as described before (21). Briefly, after fixation in 4% phosphate-buffered formalin, lung samples were marked with tissue-marking dye (General Data Company Inc, Cincinnati, OH, USA) and embedded in paraffin prior to processing several samples in one tissue cassette. H&E staining was applied to assess basic histomorphology. In combination with Giemsa staining, lymphocytes and eosinophils were highlighted. Periodic acid–Schiff (PAS) staining was performed to visualize mucopolysaccharides. Trichrome staining was used to illustrate the collagen of the basal membrane (colored blue) which allows measurement of the thickness (in μm) of the respiratory epithelium in subsegmental bronchi. All stained slides were completely scanned using the Aperio GT 450 DX whole slide image (WSI) scanner (Leica Mikrosysteme Vertrieb GmbH, Wetzlar, Germany) at 40x scan magnification with a resolution of 0.26 µm/pixel. Subsequently, all analyses of the digitized slides were performed using the Aperio Imagescope software (Leica Mikrosysteme Vertrieb GmbH, Wetzlar, Germany) and NIS Elements (Nikon CEE GmbH, Vienna, Austria) by an investigator blinded to the mouse groups.

### Immunohistochemistry/immunofluorescence and confocal microscopy

For immunofluorescence staining, 4-μm sections mounted on glass slides were deparaffinized in xylene and rehydrated in decreasing concentrations of ethanol (100–70%). Antigen retrieval was performed in 1x citrate buffer (IHC Select Citrate Buffer pH 6.0, 10x, #21545, Merck Millipore) for 20 minutes in a steamer, followed by 15 minutes at RT. After 3 washes with PBS, tissue slices were surrounded with a Hydrophobic Barrier Pap Pen (#R3777, Thermo Fisher Scientific). Samples were permeabilized with 0.1% Triton-X100 (Sigma-Aldrich) and blocked in 2% (w/v) BSA (Sigma-Aldrich) plus 5% (v/v) donkey serum (RRID: AB_2810235, Sigma-Aldrich) diluted in 1x Fish Gelatin Blocking Agent (#22010, Biotium) for 1 hour at RT. Primary antibody incubation was performed overnight at RT. The following primary antibodies were used: ASC/TMS1 (D2W8U) Rabbit mAb (1:200, RRID: AB_2799736, Cell Signaling Technology), Goat Anti-Mouse CD45 Polyclonal antibody (1:100, RRID: AB_442146, R&D Systems). On the next day, samples were washed with PBS and incubated for 2 hours at RT in the dark with secondary antibodies and the nucleus was counterstained with 4′,6-Diamidino-2-phenyl-indol-dihydrochlorid (DAPI, 1:2000, #MBD0015, Sigma-Aldrich). The following secondary antibodies were used: Donkey anti-Rabbit IgG (H+L) Highly Cross-Adsorbed Secondary Antibody, Alexa Fluor™ 568 (1:1000, RRID: AB_2534017, Invitrogen) and Donkey anti-Goat IgG (H+L) Cross-Adsorbed Secondary Antibody, Alexa Fluor™ 647 (1:1000, RRID: AB_2535864 Invitrogen). After washing again with PBS, autofluorescence was quenched by treatment with TrueBlack (#23007, Biotium) for 3 minutes according to the manufacturer’s instructions. Tissue sections were extensively washed with PBS and mounted with a microscope glass cover in ProLong Gold Antifade Mountant (Invitrogen #P36934).

As positive control for NLRP3 inflammasome formation, we analyzed ASC speck formation in BMDCs/BMDMs and digested lung tissue of healthy mice upon stimulation with LPS and Nigericin. For BMDCs/BMDMs, bone marrow was isolated from femurs and tibias of C57BL/6 mice using a 0.4×50mm needle. The cell suspension was transferred to a new tube through a 70-µm cell strainer and centrifuged for 7 minutes at 300 × g. After two washing steps with PBS, cells were taken up in BMDC medium (RPMI-1640, 10% (v/v) FCS heat-inactivated, 2 mM L-Glutamine, 100 U/ml Penicillin, 100 µg/ml Streptomycin, 50 µM 2-Mercaptoethanol) supplemented with 20 ng/ml GM-CSF, counted and 1×10^7^ cells in 4 ml seeded to 6-well plates. After two days of incubation at 37°C, 5% CO_2_, half of the medium was replaced with fresh medium containing 40 ng/mL GM-CSF. Medium was completely exchanged on day 3 and cells harvested after 6 days of differentiation and frozen in 80% BMDC medium, 10% additional heat-inactivated FCS and 10% DMSO first at -70°C and afterwards in liquid nitrogen. After thawing the cells, they were washed 3 times with BMDC medium without antibiotics and 2-Mercaptoethanol, counted, seeded at a concentration of 1×10^6^ cells/24-well on 0.01% Poly-L-Lysine-coated glass cover slips and left to recover at 37°C, 5% CO_2_ overnight.

Mouse lung cell suspension was generated by digesting lung tissue as described before (24, 25). Briefly, lung lobes were minced in RPMI containing 0.125 mg/ml Liberase™ (Roche), 0.25 mg/ml Liberase^TL^ (Roche), 0.03 mg/ml DNAse I (Sigma Aldrich) and 1 mg/ml Hyaluronidase (Sigma Aldrich) and digested for 2x30 min, 300 rpm, 37°C. Single cell suspension was seeded on 0.01% Poly-L-Lysine-coated glass cover slips.

BMDCs/BMDMs and lung cells were stimulated with 100 ng/ml LPS for 3 hours prior to stimulation with 10 µM Nigericin. The plate was briefly centrifuged (800 rpm, 10 seconds) and incubated for 45 minutes at 37°C to allow inflammasome formation. Subsequently to fix cells, 800 µl of medium was replaced with 4% paraformaldehyde to a final concentration of 3.2% for 15 minutes at RT. After washing three times with PBS, cells were permeabilized using 0.1% Triton-X100 (Sigma-Aldrich) for 10 minutes, followed by two washes with PBS. Unspecific binding sites were blocked with 2% BSA (Sigma-Aldrich) and 5% donkey serum (RRID: AB_2810235, Sigma-Aldrich) for 1 hour at RT prior to incubation with primary ASC/TMS1 (D2W8U) Rabbit mAb (1:200, RRID: AB_2799736, Cell Signaling Technology) and Goat Anti-Mouse CD45 Polyclonal antibody, (1:100, RRID: AB_442146, R&D Systems) overnight at 4°C. Afterwards, samples were extensively washed with PBS and incubated for 2 hours at RT in the dark with secondary antibodies Donkey anti-Rabbit IgG (H+L) Highly Cross-Adsorbed Secondary Antibody, Alexa Fluor™ 568 (1:1000, RRID: AB_2534017, Invitrogen), Donkey anti-Goat IgG (H+L) Cross-Adsorbed Secondary Antibody, Alexa Fluor™ 647 (1:1000, RRID: AB_2535864 Invitrogen) and the nucleus was counterstained with 4′,6-Diamidino-2-phenyl-indol-dihydrochlorid (DAPI 1:2000, MBD0015, Sigma-Aldrich). After washing the samples three times with PBS, glass cover slips were semi-dry mounted in ProLong Gold Antifade Mountant (Invitrogen #P36934) on a microscope slide.

Samples were analyzed using a Zeiss Observer Z1 fluorescence microscope equipped with an Abberior Instruments STEDYCON unit enabling widefield epifluorescence and confocal and super-resolution STED microscopy. Representative confocal images from single focal z-planes were taken using the 100× oil objective lens (NA 1.46) to visualize and investigate the ASC staining pattern in lung epithelial and immune cells. For quantification of ASC and CD45 positive cells (ASC^+^/CD45^+^) in lung tissue, 1 widefield image of mouse lung sections from 3 different mice per experimental group (n=3/group) was taken using a 20x objective lens. The number of ASC^+^/CD45^+^ cells per image was manually counted using the cell counter plugin from Fiji (ImageJ 1.53t). For image acquisition and image export, the ZEN 2 blue edition (Zeiss) or the STEDYCON Web interface software (Abberior) was used. Images were post-processed with the image processing software Fiji (ImageJ 1.53t) and Microsoft PowerPoint.

### Statistical analysis

Data were analyzed using Flow Jo 10, ProcartaPlex Analyst and GraphPad Prism 10.1.1. Statistical differences were examined using One-Way ANOVA with Tukey’s *post hoc* test, *p<0.05, **p<0.01, ***p<0.001, ****p<0.0001.

## Results

### Intranasal, adjuvant-free sensitization with BPE results in robust systemic and local allergic inflammation

Commonly used mouse models of allergic sensitization often depart from physiological relevance because they involve intraperitoneal (i.p.) or subcutaneous injection of antigens usually adsorbed to an adjuvant. Therefore, we first developed an adjuvant-free intranasal (i.n.) model of allergic lung inflammation. We then used this model to induce an immune response to BPE and to investigate the role of NLRP3 in BPE-induced allergic inflammation.

To investigate whether our i.n. sensitization model induces similar levels of allergic inflammation as commonly used adjuvant-free i.p. protocols, C57BL/6 mice were sensitized with BPE or mock-treated over a course of six weeks via i.p. or i.n. according to the scheme shown in **Figure 1A**. One week after the last immunization, all groups were challenged i.n. with BPE on three consecutive days, followed by the isolation of serum, broncho-alveolar lavage fluid (BALF), lung tissue and restimulation of splenocytes after another 24 hours.

Both i.p.- and i.n.-sensitized mice showed slightly higher serum IgG titers than mock-treated animals but barely produced Th1-associated IgG2c. Strikingly, however, the Th2-related antibody subtypes IgG1 and particularly IgE were significantly higher in the i.n. group (**Figure 1B**).

Restimulation of splenocytes with BPE revealed high amounts of Th1-associated cytokines and chemokines in all groups (**Figure 1C**), which can be explained by the considerable amounts of bacterial PAMPs, as described by McKenna and colleagues (8), in the pollen extract that we used. Remarkably, Th2-associated cytokines, in particular IL-4, IL-5 and IL-13, were only produced by BPE-sensitized mice but not by mock-treated animals via both sensitization routes (**Figure 1C**; **Supplementary Figure 1A**). This set of data indicates that sensitization via the i.n. route induces robust BPE-specific, Th2-associated systemic inflammation.

**Figure 2:**
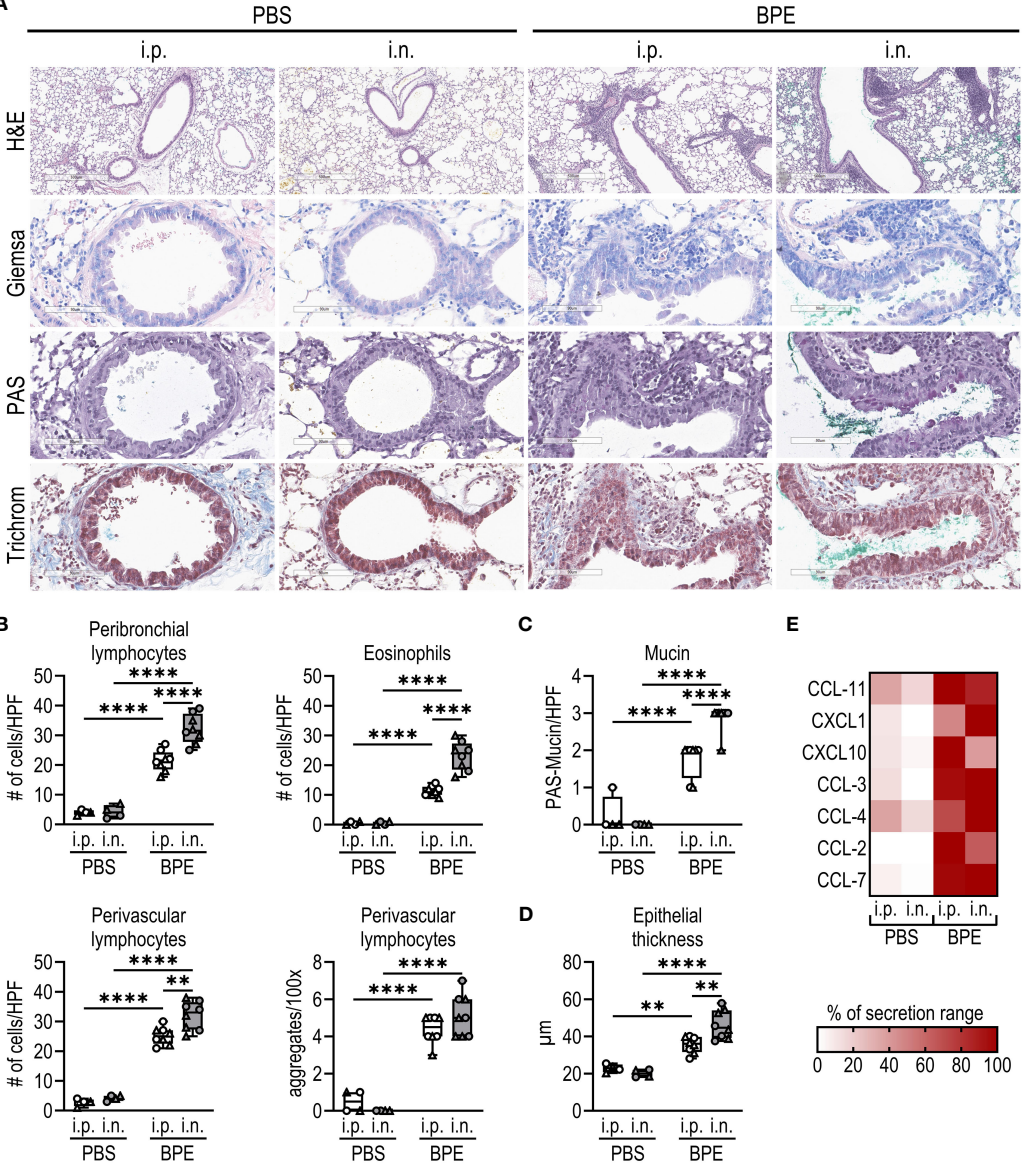
I.n. sensitization results in robust allergic lung inflammation. **(A–D)** Fixed lung tissue sections of C57BL/6 WT mice (**Figure 1**) were stained with **(A)** H&E, Giemsa, PAS and trichrome (H&E with magnification of 5.2x (bar at 500 µm), all other stainings with magnification of 25.6x (bar at 90 µm)). **(B)** Lymphocytes infiltrating the peribronchial or perivascular space and eosinophils were quantified as counts per HPF. Perivascular lymphocyte aggregates were counted per 100x. **(C)** Mucin production per HPF and **(D)** epithelial thickness were evaluated from stained lung sections. Box plots show median of 4–8 mice per group of two individual experiments with different batches of pollen extract. Each mouse is depicted as one data point. **(E)** Chemokine levels in broncho-alveolar lavage fluid were determined by Luminex Multiplex technology. Medians of each group are shown as % of maximum secretion. HPF, high power field. To analyze statistically significant differences, one-way ANOVA with Tukey’s post hoc test was performed. **p<0.01, ****p<0.0001.

As BP is an inhalant allergen, we also studied local lung inflammation by histological staining of fixed tissue sections and analyzed cell and cytokine composition in BALF. In general, sensitization with BPE resulted in highly increased cell infiltration and chemokine secretion into the lungs irrespective of the sensitization route (**Figures 2A, B, E**; **Supplementary Figures S1B, C**). However, consistent with the increase in Th2-associated antibodies, the numbers of eosinophils as well as peribronchial and perivascular lymphocytes were significantly increased upon sensitization via the nasal route compared to the i.p. route (**Figures 2A, B**). Additionally, mucin production and epithelial thickness were significantly higher after i.n. sensitization (**Figures 2C, D**). As shown in **Supplementary Figure S1C**, we also observed higher infiltration by B cells in the i.n. sensitization group, whereas similar numbers of CD4^+^ T cells and neutrophils migrated into the lungs of the i.p. and i.n. groups. These findings show that the i.n. route induces a highly allergic lung pathology.

Taken together, i.n. sensitization resulted in a robust systemic and local allergic immune response, while the i.p. route failed to induce an IgE antibody response and provoked significantly less lung inflammation, confirming the physiological relevance of our i.n. model.

### Intranasal sensitization to BPE is attenuated in *Nlrp3*-deficient animals

Based on the stronger allergic outcome of the adjuvant-free i.n. sensitization model, we used this procedure to elucidate the role of NLRP3 in BP allergy.

**Figure 3:**
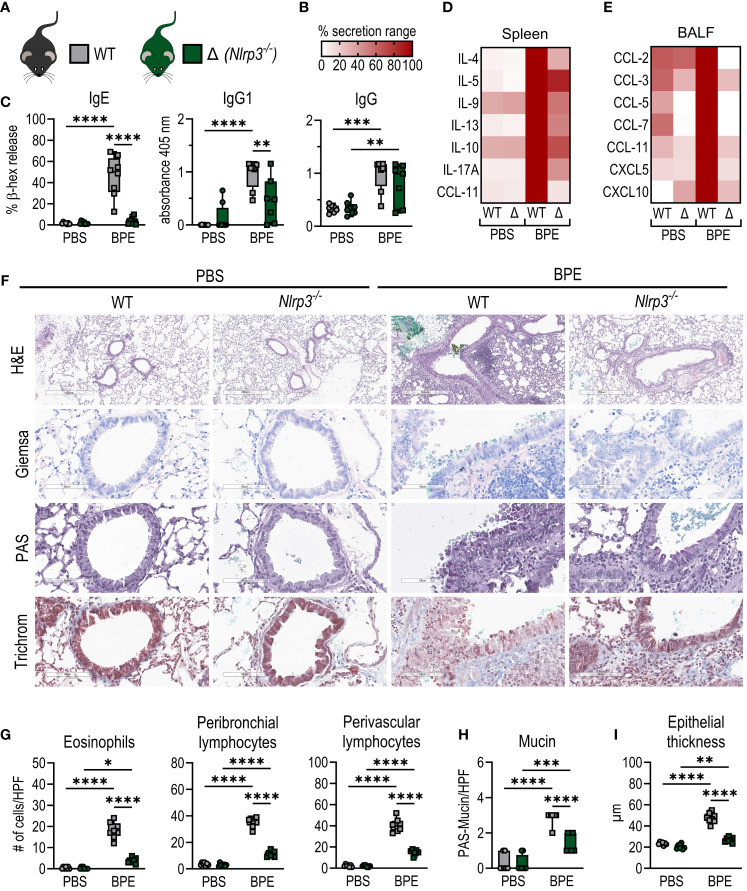
*Nlrp3-*deficient mice are partially protected from allergic sensitization. **(A)** C57BL/6 WT (grey) or *Nlrp3^-/-^* (Δ, green) mice were sensitized i.n. as shown in **Figure 1A**. **(B)** Legend for heatmaps in **(D, E)**. **(C)** BPE-specific serum IgE was measured via antigen-induced cross-linking of Fcϵ-receptors on RBL-2H3 cells and subsequent degranulation and release of β-hexosaminidase (β-hex release). IgG antibody subtypes specific to BPE were analyzed by ELISA. **(D)** Splenocytes were restimulated with BPE for 3 days and secreted cytokines and **(E)** chemokine levels in broncho-alveolar lavage fluid were measured by Luminex Multiplex technology. **(F–I)** Lung tissue sections were stained with H&E, Giemsa, PAS and trichrome to quantify **(G)** infiltrating peribronchial and perivascular lymphocytes, eosinophils, **(H)** mucin production and **(I)** epithelial thickness. H&E with magnification of 5.2x (bar at 500 µm), all other stainings with magnification of 25.6x (bar at 90 µm). **(C, G, H)** Data of three individual experiments are shown as box plots, with each data point representing one mouse. **(D, E)** Medians are normalized to the maximum release of each cytokine. BPE, birch pollen extract; HPF, high power field. To analyze statistical differences, one-way ANOVA with Tukey’s posthoc test was performed. *p<0.05, **p<0.01, ***p<0.001, ****p<0.0001.

As described before, C57BL/6 WT and *Nlrp3*-deficient mice (whole-body knockout) were mock-treated or sensitized i.n. with BPE over a course of 6 weeks. Interestingly, after three days of challenge with BPE, serum-IgG1 and -IgE levels were significantly decreased in sensitized *Nlrp3^-/-^* mice compared to WT (**Figure 3C**). In line with this, cytokine secretion by restimulated splenocytes was strongly attenuated in *Nlrp3*-deficient animals (**Figure 3D**, **Supplementary Figure 2A**), indicative of a significantly reduced systemic response to BPE in *Nlrp3^-/-^* mice.

Consistent with these findings, *Nlrp3*-deficient animals showed a massive decrease in local lung inflammation, characterized by lower or significantly less chemokine levels in BALF (**Figure 3E**; **Supplementary Figure 2B**) and reduced infiltration by eosinophils, B cells and CD4^+^ and CD8^+^ T cells compared to WT mice (**Figures 3F, G**; **Supplementary Figure 2C**). Additionally, we observed significantly attenuated signs of allergic inflammation, including reduced lymphocyte accumulation in the perivascular space, mucin production and epithelial thickening (**Figures 3H, I**; **Supplementary Figure 2D**).

These data clearly show significantly reduced signs of systemic and local allergic inflammation in *Nlrp3*-deficient mice compared to WT animals, suggesting that the absence of NLRP3 partially protects against allergic sensitization to BP and raising the question of the specific role of NLRP3 in this process.

### Generation of a BPE-specific Th2 response is not dependent on inflammasome-induced caspase 1 activation

Because NLRP3 is well described as an inflammasome-forming NLR but has also been described to act as a transcription factor, we next asked whether the induction of BP allergy in our sensitization model depends on NLRP3 as an inflammasome driver.

**Figure 4:**
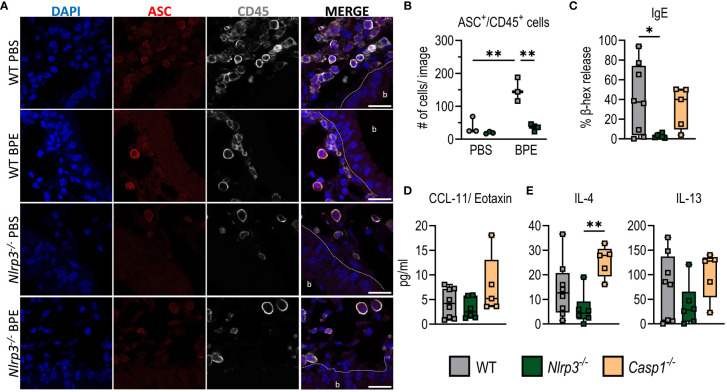
Allergic sensitization to BPE is independent of inflammasome-induced caspase 1 activation. **(A)** Immunofluorescence staining of lung sections from C57BL/6 and *Nlrp3^-/-^* mice (**Figure 3**) against ASC (red) and CD45 (white). Dapi (blue) was used to stain cell nuclei. Scale bar is 20 µm. The dashed line marks the epithelial cell barrier. b= bronchiole. **(B)** Quantification of ASC^+^/CD45^+^ cells of 3 mice/group, 1 image/mouse as depicted in **(A)**. **(C)** BPE-specific serum IgE was measured via antigen-induced cross-linking of Fcϵ-receptors on RBL-2H3 cells and subsequent degranulation and release of β-hexosaminidase (β-hex release). **(D, E)** Cytokines secreted into BALF **(D)** or by restimulated splenocytes **(E)** were measured by Multiplex technology. The data are presented as box plots of at least 5 mice per treatment group of 3 individual experiments. Each mouse is depicted as one data point. One-way ANOVA with Tukey’s post hoc test was performed to determine statistical significance. *p<0.05, **p<0.01.

Immunofluorescence staining as a method to detect NLRP3 expression has serious disadvantages, as available NLRP3 antibodies very often also bind non-specifically. However, since NLRP3 activation leads to the oligomerization of ASC proteins, the so-called ASC pyroptosome (26), we analyzed ASC-speck formation in lung sections of C57BL/6 and *Nlrp3^-/-^* mice as a surrogate for inflammasome activation. ASC was significantly higher expressed in lung tissue of sensitized WT animals and evenly distributed within CD45^+^ immune cells irrespective of genotype. However, even after three days of BPE challenge, no ASC-specks were observed in CD45^+^ cells within the lungs of sensitized mice (**Figures 4A, B**), whereas BMDCs/BMDMs and CD45^+^ cells of healthy digested lungs treated with LPS and nigericin, used as positive control, showed clear ASC-speck formation (**Supplementary Figure 3A**). The fact that lung epithelial cells also did not show ASC-speck formation suggests that inflammasome formation may play only a minor role at best in these particular cells in BPE-induced allergic inflammation.

To further analyze the potential inflammasome-independent role of NLRP3 in BP allergy, we next sensitized *Casp1*-deficient animals in addition to C57BL/6 WT and *Nlrp3^-/-^* mice as described before. Interestingly, absence of Caspase 1 did not resemble NLRP3 deficiency. Instead, *Casp1^-/-^* mice showed amounts of serum IgE and IgG1, IL-4-, IL-5- and IL-13-secretion by splenocytes, and CXCL1/CCL11 in the lungs similar to those of the WT control group (**Figures 4C–E**; **Supplementary Figures 3B–D**). This suggests that Caspase 1 deficiency does not result in reduced allergic inflammation. In addition, we did not observe a significant increase in IL-1β secretion by splenocytes or increased IL-1β levels in the BALF (**Supplementary Figures 3C, D**) after sensitization with BPE in either WT, Nlrp3^-/-^ or Casp1^-/-^ mice, further confirming that NLRP3 inflammasome formation is dispensable in BPE-induced allergic responses.

### Allergic sensitization to BPE requires the presence of NLRP3 in the hematopoietic system

**Figure 5:**
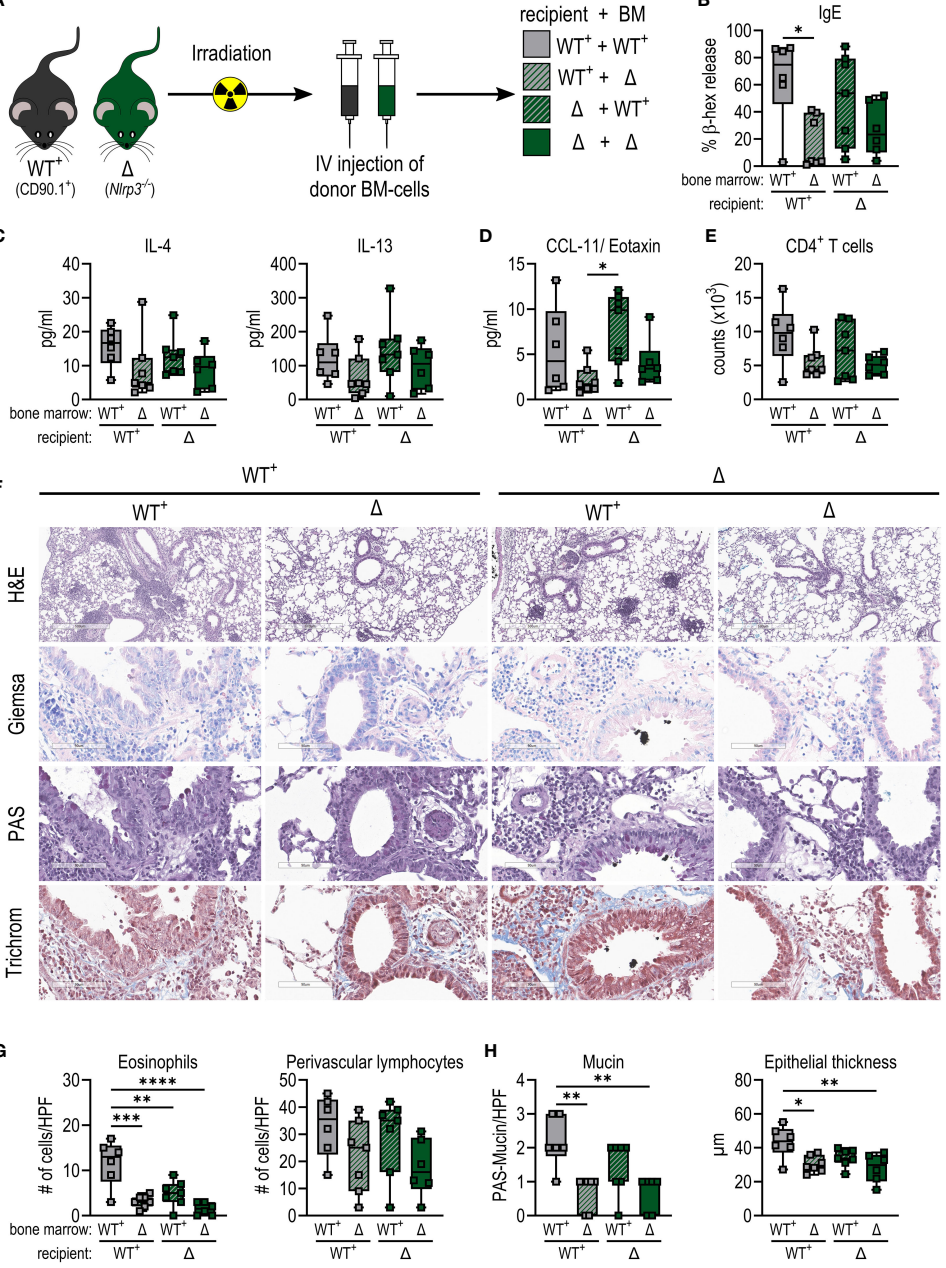
Allergic sensitization to BPE depends on the presence of NLRP3 in the hematopoietic system rather than in the epithelium. **(A)** WT^+^ (CD90.1^+^) and (*Nlrp3^-/-^* CD90.2^+^) mice were irradiated and received bone marrow (BM) from one of the two strains. After successful engraftment, chimeras were sensitized as shown before. **(B)** Serum IgE detected by a cell-based assay. **(C, D)** Cytokines secreted by splenocytes **(C)** or into BALF **(D)** measured by Multiplex technology. **(E)** Infiltration by CD4^+^ T cells into BALF determined by flow cytometry. **(F–H)** Lung tissue sections were stained with H&E, Giemsa, PAS and trichrome (H&E with magnification of 5.2x (bar at 500 µm), all other stainings with magnification of 25.6x (bar at 90 µm)) to quantify **(G)** infiltrating eosinophils and lymphocytes and **(H)** mucin production and epithelial thickness. Box plots show median of at least 6 mice per treatment group of 3 individual experiments. Each mouse is depicted as one data point. One-way ANOVA with Tukey’s post hoc test was performed to determine statistical significance. *p<0.05, **p<0.01, ***p<0.001, ****p<0.0001.

To understand whether NLRP3 exerts its key function during allergic sensitization primarily in hematopoietic cells that migrate to the lung or rather in the lung epithelium, we irradiated C57BL/6- CD90.1 (WT^+^) and *Nlrp3^-/–^* CD90.2 mice and reconstituted their hematopoietic system with bone marrow from either the same or the other mouse strain (**Figure 5A**). In both models, we achieved high engraftment rates, with 98–99% of donor cells after engraftment of *Nlrp3*-deficient bone marrow and 58–92% of donor cells after engraftment of WT^+^-cells (**Supplementary Figure 3E**).

After BPE sensitization and challenge, mice lacking NLRP3 in the hematopoietic system showed reduced IgE levels compared to animals receiving WT bone marrow (**Figure 5B**). In line with this, enhanced levels of IL-4 and IL-13 secreted by restimulated splenocytes and CCL11 in the lungs were observed in mice expressing NLRP3 in the hematopoietic system, compared to animals expressing NLRP3 only in the epithelium (**Figures 5C, D**).

Consistent with these findings, in the absence of NLRP3 in the hematopoietic compartment, animals showed reduced levels of infiltration by eosinophils and lymphocytes, in particular CD4^+^ T cells, into the lungs and perivascular areas (**Figures 5E–G**; **Supplementary Figure 3G**). In addition, mucin production and epithelial thickness were slightly higher in mice expressing NLRP3 in immune cells compared to a deficiency of NLRP3 in the hematopoietic system (**Figures 5F, H**).

Taken together, these findings imply that while NLRP3 inflammasome formation and subsequent activation of Caspase 1 is dispensable in our model of BP allergy, NLRP3 expression in the hematopoietic system is essential for BPE-induced allergic inflammation.

## Discussion

Allergic reactions to BP or other inhalant allergens are increasing in the northern hemisphere, which needs to be met by a thorough understanding of the sensitization process. It has become clear that the pollen context, which includes a whole microbiome among other factors, plays a crucial part in the induction of allergies. The bacterial patterns associated with the microbiome have been shown to be capable of activating the NLRP3 inflammasome (11). However, the specific role of NLRP3 in allergic asthma remains controversial.

Although numerous studies have focused on allergic processes in the lung, established intranasal (i.n.) sensitization protocols (27, 28) have mostly been replaced by less laborious models such as intraperitoneal (i.p.) injection of allergen or allergen extracts in combination with adjuvants such as alum. While these convenient procedures are adequate to examine allergic responses in general, they are insufficient for investigating the sensitization process to inhaled allergens such as BP. Furthermore, the fact that alum mediates its adjuvant function via activation of the NLRP3 inflammasome (29, 30) makes it impossible to clearly distinguish alum-induced immune responses from direct responses to the allergen or allergen extract.

Therefore, we aimed to develop a physiologic and adjuvant-free mouse model to study the involvement of NLRP3 in BP allergy.

Hence, we first compared allergic immune reactions in C57BL/6 WT mice in response to either i.n. or i.p. administration of BPE without the use of adjuvants. After 6 weeks of sensitization and 3 days of challenge, significantly higher allergen-specific IgE and IgG1 antibody responses and local lung inflammation were detected in the i.n. group, demonstrating the power of this route of sensitization.

I.n. allergic sensitization has already been described with other allergen sources, including Timothy grass pollen (31, 32) and Japanese cedar (33). The reported sensitization procedures, including the one we used in this study, share a protocol of repeated administration of antigen via the i.n. route twice per week, although the ultimate duration differs between 6 and 15 weeks. In all studies, increased production of Th2-associated antibodies and cell infiltration into the lung were observed. An abbreviated model of ragweed pollen allergy comprising 16 administrations over a course of 18 days (34) as well as administration of house dust mite (HDM) allergen via the i.n. but not via the oral route (35) induced specific IgE and lung infiltration by lymphocytes, which also resembles our findings. Sufficient induction of systemic and local allergic inflammation was observed with a BPE-model of shorter duration than ours, probably because it used a much higher dose of protein (250 µg) per i.n. instillation every 2–4 days over a period of almost 3 weeks (36). Even an i.n. infection model with the parasite *Leishmania major* shows a substantial Th2 response, whereas subcutaneous infection induced predominantly Th1 responses (37). The importance of the specific sensitization route is also evident in humans, as nasal exposure to grass or BP allergens boosted serum IgE levels in allergic patients while dermal contact did not result in a relevant increase (38). Taking the results of these studies together, it is clear that the pulmonary mucosa targeted by i.n. protocols plays an essential role in the natural sensitization process to various allergens, thus highlighting the importance of more physiologic sensitization protocols for the investigation of allergic sensitization.

We used a highly relevant i.n. model to study the role of NLRP3 in allergic sensitization to BP. Strikingly, compared to WT, *Nlrp3*-deficient mice displayed significantly attenuated type-2 responses both at the systemic level (IgE/IgG1, Th2-associated cytokine secretion of splenocytes) and locally in the lung (cell infiltration, lung pathology, chemokines in BALF) (**Figure 3**). While some research groups have reported similar findings in ovalbumin-induced models of NLRP3-dependent allergic inflammation (15, 16, 29), other groups have not seen any differences in *Nlrp3^-/-^* mice (17, 18). We suggest that these contradictory findings might be a result of differences in the sensitization route and antigen used. By comparing the different procedures, it is evident that sensitization via the i.p. route is usually independent of NLRP3 (17, 18), whereas NLRP3 does play a role in sensitization via the skin (15, 16). Additionally, the type of allergen source appears to be highly consequential in the development of allergies. For example, the major group 1 allergens from HDM (Der p 1/Der f 1) are particularly immunogenic (39), whereas Bet v 1 does not induce allergic sensitization by itself but requires the whole BP context (9). This might explain why with HDM-induced models, *Nlrp3*-deficient mice show similar signs of allergy as their WT counterparts (17, 18). Overall, these studies indicate that NLRP3 might be dispensable for other inhalant allergens, but, as we showed here, it is involved in inducing Th2-responses against BP.

Similar to what has been demonstrated by Bruchard et al. (16) in an ovalbumin-induced model, only *Nlrp3*- but not *Casp1*-deficient animals showed attenuated signs of allergy, suggesting an inflammasome-independent role of NLRP3 during sensitization to BP as well. This is also supported by the fact that we did not observe ASC-speck formation in lung tissue (**Figure 4**).

Although conducted in BALB/c mice, it has been shown that functional IL-1β signaling via NLRP3 is required in an ovalbumin-induced model of allergy. Interestingly, Anakinra, a potent inhibitor of IL-1β, was able to suppress eosinophil infiltration into the lungs when administered during challenge, but not when injected during the sensitization phase (40). A similar outcome was observed by Ma and colleagues (41) in an HDM-induced model. Here, a less-known NLRP3 inhibitor, RRx-001, was able to significantly reduce cell infiltration into the lungs. Injection of MCC950, one of the most commonly used NLRP3 inflammasome inhibitors, during the challenge phase showed a therapeutic effect in an OVA-induced model in C57BL/6 mice as well (fewer eosinophils/goblet cells, reduced IgE levels) (42). In another study, after sensitization with OVA, mice were challenged for 7 days additionally to nasal administration of MCC950, which resulted in significantly less IgE and cell infiltration into the lungs (43). These findings suggest an ameliorative effect of NLRP3 inhibition during acute challenge, possibly since the initial sensitization to OVA occurred via usage of alum and therefore via activation of NLRP3. However, we showed that NLRP3 inflammasome and subsequent Caspase 1 activation are not required for sensitization to BPE via the i.n. route.

Unfortunately, there is yet no mouse line commercially available to target NLRP3 in a cell type-specific manner, which is why we generated bone marrow chimeras expressing NLRP3 either in the epithelium or in the hematopoietic compartment. After successful engraftment, mice were again sensitized and challenged as described before. While NLRP3 was dispensable in the epithelium, it was required in the bone marrow to induce a similar Th2 response against BP as in WT mice, supporting our hypothesis that NLRP3 is essential in the hematopoietic system. However, to really understand the role of NLRP3 in BP allergy as well as in other allergic disorders, a cell type-specific knockout or knock-in similar to what has already been established for the NAIP/NLRC4 inflammasome (44) would be beneficial.

In conclusion, this study shows that NLRP3 plays a key role in BPE-induced allergic inflammation. Although the formation of the NLRP3 inflammasome may play a minor role in this context, the presence of NLRP3, especially in the CD45^+^ hematopoietic compartment, is crucial for the induction of a strong allergic response to BPE via the i.n. route.

## Data availability statement

The original contributions presented in the study are included in the article/**Supplementary Material**. Further inquiries can be directed to the corresponding author/s.

## Ethics statement

The animal study was approved by the Austrian Federal Ministry of Education, Science and Research (permit numbers: 66.012/0015-V/3b/2019 and 2021-0.588.488). The study was conducted in accordance with the local legislation and institutional requirements.

## Author contributions

RB: Writing – original draft, Conceptualization, Data curation, Formal analysis, Investigation, Methodology, Visualization, Writing – review & editing, Validation. H-HD: Writing – original draft, Data curation, Investigation, Methodology, Formal analysis, Validation. DN: Writing – original draft, Data curation, Visualization, Formal analysis, Investigation, Methodology, Validation. MU: Writing – original draft, Data curation, Investigation, Visualization, Formal analysis, Methodology. TN: Writing – original draft, Supervision, Writing – review & editing, Conceptualization. MJ: Writing – review & editing, Investigation. AT: Writing – review & editing, Investigation. HS: Writing – review & editing, Investigation. IG: Writing – review & editing, Resources, Conceptualization. RW: Writing – review & editing, Conceptualization, Resources, Methodology. AS: Writing – review & editing, Investigation, Resources, Conceptualization, Methodology, Supervision. JH-H: Writing – original draft, Writing – review & editing, Conceptualization, Funding acquisition, Project administration, Resources, Supervision.

## Supplementary material

Supplementary Figure 1 Systemic and local immune responses to BPE induced by different sensitization routes in C57BL/6 WT mice. **(A)** Systemic response and **(B–D)** local allergic lung inflammation of C57BL/6 mice sensitized via i.p. or i.n. administration of BPE. **(A)** Splenocytes were restimulated with BPE for 3 days and cytokine secretion was analyzed via Multiplex technology. **(B)** Chemokines secreted into the BALF were quantified using Multiplex technology. **(C)** Cell infiltration into the lung was measured by flow cytometry. Box plots show median of 4–8 mice per treatment group of two individual experiments with different batches of pollen extract. Each mouse is depicted as one data point. One-way ANOVA with Tukey’s post hoc test was performed to determine statistical significance. *p<0.05, **p<0.01.

Supplementary Figure 2 Systemic and local immune responses to BPE-sensitization are reduced in *Nlrp3^-/-^* mice. C57BL/6 WT (grey) or *Nlrp3^-/-^* (Δ, green) mice were sensitized i.n. as shown in **Figure 1A**. **(A)** Splenocytes were restimulated with BPE for 3 days and secreted cytokines and **(B)** chemokine levels in broncho-alveolar lavage fluid were measured by Luminex Multiplex technology. **(C)** Cell infiltration into the lung was measured by flow cytometry. **(D)** Accumulation of lymphocytes in perivascular spaces of the lung were counted as number of aggregates per 100×. Data of three individual experiments are shown as box plots, with each data point representing one mouse. To analyze statistical differences, one-way ANOVA with Tukey’s post hoc test was performed. *p<0.05, **p<0.01, ***p<0.001, ****p<0.0001.

Supplementary Figure 3 Allergic sensitization to BPE is independent of inflammasome-induced caspase 1 activation and depends on the presence of NLRP3 in the hematopoietic system. **(A)** Positive control for NLRP3 inflammasome activation in BMDCs/BMDMs and digested lung tissue determined by immunofluorescence staining of ASC specks. Antibody staining and specificity were tested by omitting of primary antibody (negative control). Scale bar is 20 µm. **(B)** Serum IgG1 measured by ELISA. **(C, D)** Cytokines secreted by splenocytes **(C)** or into BALF **(D)** measured by Multiplex technology. **(E)** Percentage of engraftment determined by flow cytometric analysis of the number of CD90.1^+^ or CD90.2^+^ cells in blood or in BALF. **(F)** Serum IgG1 measured by ELISA and cytokines secreted by splenocytes measured by Multiplex technology. **(G)** Number of peribronchial lymphocytes and perivascular lymphocyte aggregates. Box plots **(A–C, F, G)** show median of at least 5 mice per treatment group of 3 individual experiments. Each mouse is depicted as one data point. Bar graphs **(E)** show mean ± SD. One-way ANOVA with Tukey’s post hoc test was performed to determine statistical significance. *p<0.05, **p<0.01, ***p<0.001.
