# Supplementary figures and images for: NLRP3 promotes allergic responses to birch pollen extract in a model of intranasal sensitization
Source: Front Immunol. 2024 Jun 12;15:1393819. doi: 10.3389/fimmu.2024.1393819 (PMC11199694; doi:10.3389/fimmu.2024.1393819)

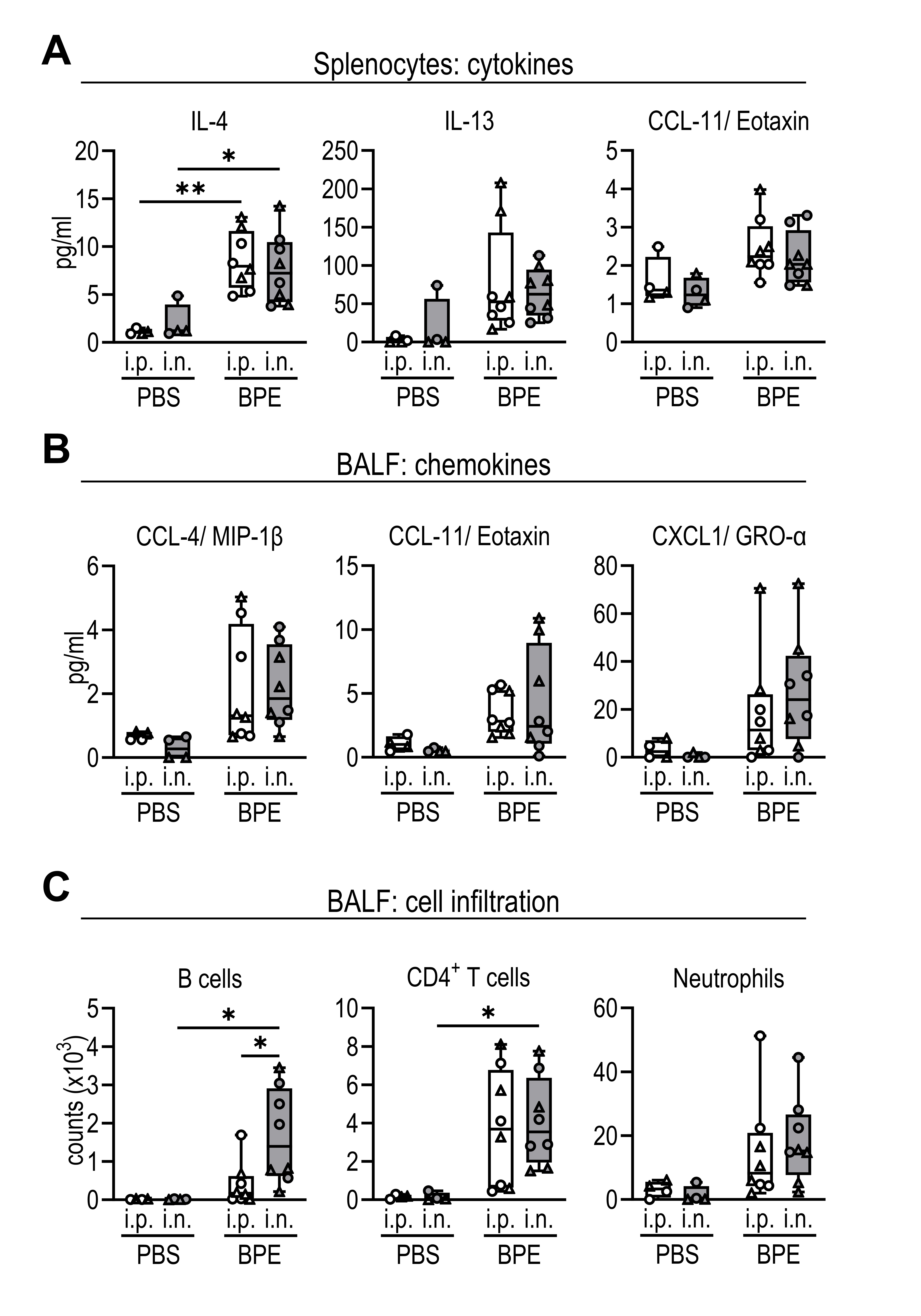

Supplement: Supplementary Figure 1 — Systemic and local immune responses to BPE induced by different sensitization routes in C57BL/6 WT mice. (A) Systemic response and (B–D) local allergic lung inflammation of C57BL/6 mice sensitized via i.p. or i.n. administration of BPE. (A) Splenocytes were restimulated with BPE for 3 days and cytokine secretion was analyzed via Multiplex technology. (B) Chemokines secreted into the BALF were quantified using Multiplex technology. (C) Cell infiltration into the lung was measured by flow cytometry. Box plots show median of 4–8 mice per treatment group of two individual experiments with different batches of pollen extract. Each mouse is depicted as one data point. One-way ANOVA with Tukey’s post hoc test was performed to determine statistical significance. *p<0.05, **p<0.01. [file Image_1.jpg]
